# Multi-omics insights into the mechanisms and prognosis of IPF

**DOI:** 10.1186/s41021-026-00364-0

**Published:** 2026-08-17

**Authors:** Dong Chen, Qianwei Chen, Honghui Liu, Zhoutong Wang, Xinyu Liu, Juan Hu, Chao Lu

**Affiliations:** 1 Joint Research Centre for Occupational Medicine and Health of IHM, Anhui University of Science and Technology, Huainan, China; 2 The First Hospital of Anhui University of Science and Technology, Huai Nan, China; 3 Lianyungang Second People’s Hospital Affiliated to Kangda, College of Nanjing Medical University, Lianyungang, China

**Keywords:** Idiopathic pulmonary fibrosis, IPF, Multi-omics, Genetic susceptibility, Precision medicine, Epigenetics, Gene-environment interaction

## Abstract

Idiopathic pulmonary fibrosis (IPF) represents another progressive and devastating interstitial lung disease found across populations, with a prominent male predominance accounting for approximately 70% of cases. Regional variation in IPF-related genetic polymorphisms, especially in the Americas, Europe, and Asia, points to a hereditary basis. Family history is the main risk factor. Rare mutations may alter outcomes through gene interactions. Environmental factors such as smoking can worsen genetic risk by epigenetic mechanisms like DNA methylation. Unlike studies that focus narrowly on specific genetic variants or processes, this study integrates the genetic variation map, multi-omics regulatory networks, and the effects of environmental exposures in IPF. This links clinical decision-making and gene discovery. This study examines mechanisms, therapies, and prognoses to clarify the impact of epigenetics and genetics on disease development. It also proposes using a multi-gene risk score (MGRS) to predict outcomes and design targeted treatments. The research advances the translation of basic knowledge into therapies. Theoretical and clinical support can aid doctors treating IPF.

**Clinical trial number**

Not applicable.

## Introduction

### Global burden of disease and clinical challenges

Idiopathic Pulmonary Fibrosis (IPF) is a long-term, worsening, and fibrotic form of interstitial pneumonia. The cause of this disease remains unclear. Its characteristics include irreversible scarring and extensive remodeling of lung tissue, which subsequently lead to destruction of lung structure and failure of gas exchange [1, 2]. This disease is closely related to age and is particularly prevalent among men over 60 years old. The average age of diagnosis is about 66 years [3]. Epidemiological data from 2023 show substantial regional differences: North America has the highest incidence and prevalence, at 9.0 and 27.2 per 100,000 population, approximately twice that of Europe (5.1/14.6) and Asia (4.4/14.8) [4]. Sex differences are also evident, with males accounting for about 70% of cases [5]. The proportion of patients receiving antifibrotic therapy was higher in males than in females (United States: 30% vs. 21.9%, *P* < 0.0001; South Korea: 80% vs. 73%) [6, 7]. Males had a poorer prognosis, with mortality in the United States 1.63 times higher than in females [8].Currently, only two anti-fibrotic drugs, pirfenidone and nintedanib, have received universal approval for the treatment of IPF [9]. Although these drugs can safely slow the disease and improve lung function, they cannot stop or reverse the disease process, nor can they significantly stabilize lung function or improve patients’ quality of life. Some patients even develop drug resistance, resulting in limited efficacy [10–12]. Lung transplantation remains the only treatment option that can potentially cure IPF patients and significantly prolong their survival period [13]. However, lung transplantation is constrained by multiple issues, such as a shortage of donors, difficulties in determining the surgical timing, and chronic dysfunction of the graft after the operation. Studies have shown that approximately 50% of recipients will experience chronic lung allograft dysfunction (CLAD) within 5 years after the transplant, and the occurrence of CLAD is closely related to the underlying cause; for instance, in cystic fibrosis patients, the abundance of *Pseudomonas aeruginosa* and its virulence metabolites in the bronchoalveolar lavage fluid increases significantly, which is highly correlated with the development of CLAD [14].

### Shifting drivers of ipf etiology from environment to genetics

In the 20th century, the main idea behind the cause of IPF was that it was due to factors in the environment and at work. As they continue their investigation into the cause of IPF, scientists are now focusing less on “environmental exposure dominance” and more on “the interaction between genetic susceptibility and environmental damage”. The major focus of the etiological theory of IPF in the twentieth century was on occupational and environmental exposure factors. This view was supported by numerous epidemiological studies. For instance, Baumgartner et al. [15] reported a significant association between IPF and factors such as metal dust, wood dust, agricultural activities, and smoking in a large-scale case-control study Taskar and Coultas’ subsequent meta-analysis quantitatively assessed these risk factors, highlighting the critical role of environmental factors in the onset of IPF [16]. Further analysis from the perspective of pathological mechanisms indicates that these inhaled harmful substances directly damage alveolar epithelial cells (AECs), initiating the fibrosis process and making it difficult to distinguish between environmental lung diseases such as asbestosis and IPF on the basis of pathology [17].In recent years, Genome-Wide Association Study (GWAS) has greatly advanced understanding of IPF’s genetic architecture, establishing a new paradigm: susceptibility is mediated by multiple common genetic variants and mechanisms like telomere maintenance, mTOR signaling, and cell proliferation [18, 19]. This updated framework proposes that specific genetic variations act as the initial trigger, sensitizing the lung epithelium—especially in the distal bronchioles and alveoli—to environmental stressors like cigarette smoke or viral infections, which serve as subsequent triggers. These factors set off a dysfunctional repair process leading to progressive fibrosis [20–22]. This gene-environment concept is strongly supported by empirical data: mutations in genes responsible for telomere maintenance, like Telomerase reverse transcriptase (*TERT*) and Telomerase RNA component (*TERC*), have been linked to IPF through cellular senescence [23]. A systematic review published in 2025 indicated that the risk allele of the single nucleotide polymorphism (rs35705950) in the promoter region of the Mucin 5B (*MUC5B*) gene significantly increased the risk of IPF, suggesting that abnormal airway mucus secretion is a significant factor in the process of interstitial fibrosis [24]. In addition, the research has successively identified several susceptibility gene loci related to the function of alveolar epithelium and cell adhesion, such as Surfactant protein A2 (*SFTPA2*), Surfactant protein C (*SFTPC*), and Surfactant protein D (*SFTPD*) [25]. Essentially, an innate genetic framework and environmental factors that cause epigenetic reprogramming interact to prod uce the pathophysiology of IPF.

### Framework and novel insights of the review

**Fig. 1:**
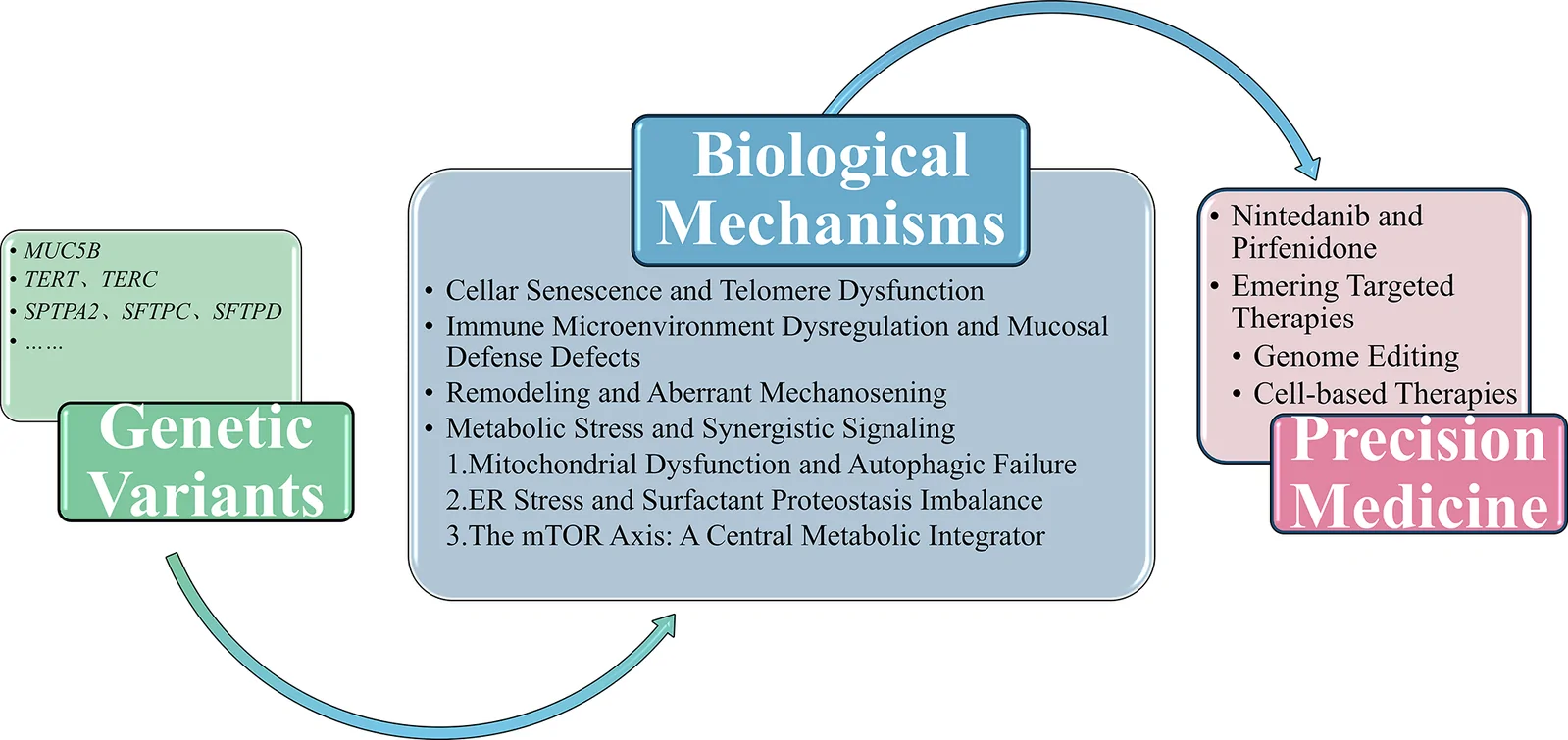
Integrated Framework of “Genetic Variation–Multi-omic Mechanisms–Precision Medicine” for IPF

The core framework starts with the exploration of IPF genetic variations. As illustrated in Fig. 1, the core framework of this review integrates molecular mechanism analysis across multiple omics levels and extends to the development of targeted therapies, efficacy prediction, and prognostic stratification throughout the entire precision diagnosis and treatment chain. Together, these form a closed-loop research route: “genetic variations-multi-omics mechanisms-precise treatment-prognostic assessment”. As genetic research on IPF enters the “post-GWAS era”, a vast amount of genetic data needs to be systematically organized, explained, and translated into clinical value. This review aims to move beyond simply listing genes. It seeks to build a complete, logical system from “genes” to “prognosis”. First, we will systematically review currently confirmed IPF-related susceptibility genes and their functions. We focus on how these genetic variations drive IPF development through biological processes. On this basis, we will deeply explore how these mechanisms provide a theoretical basis for the discovery of new drug targets, and review current clinical trials and prospects for precise treatment based on genetic mechanisms. Finally, we will discuss how genetic markers can be used to assess illness risk. Instead of focusing on a specific gene pathway or therapy development, this review provides a thorough, logical chain of events from “genetic discovery” to “clinical transformation” for IPF. Through deep integration of genetic variation maps, multi-omics regulatory networks, precise targeting strategies, and multi-gene risk score (MGRS) prognostic stratification systems, this paper not only reveals the intrinsic mechanism by which genetic susceptibility drives disease progression, but also proposes a precise medical path based on individual genetic background in a forward-looking manner, aiming to provide comprehensive theoretical guidance for clinical research and individualized diagnosis and treatment of IPF.

## Genetically driven core mechanistic clusters in IPF

### Cellular senescence and telomere dysfunction

**Fig. 2:**
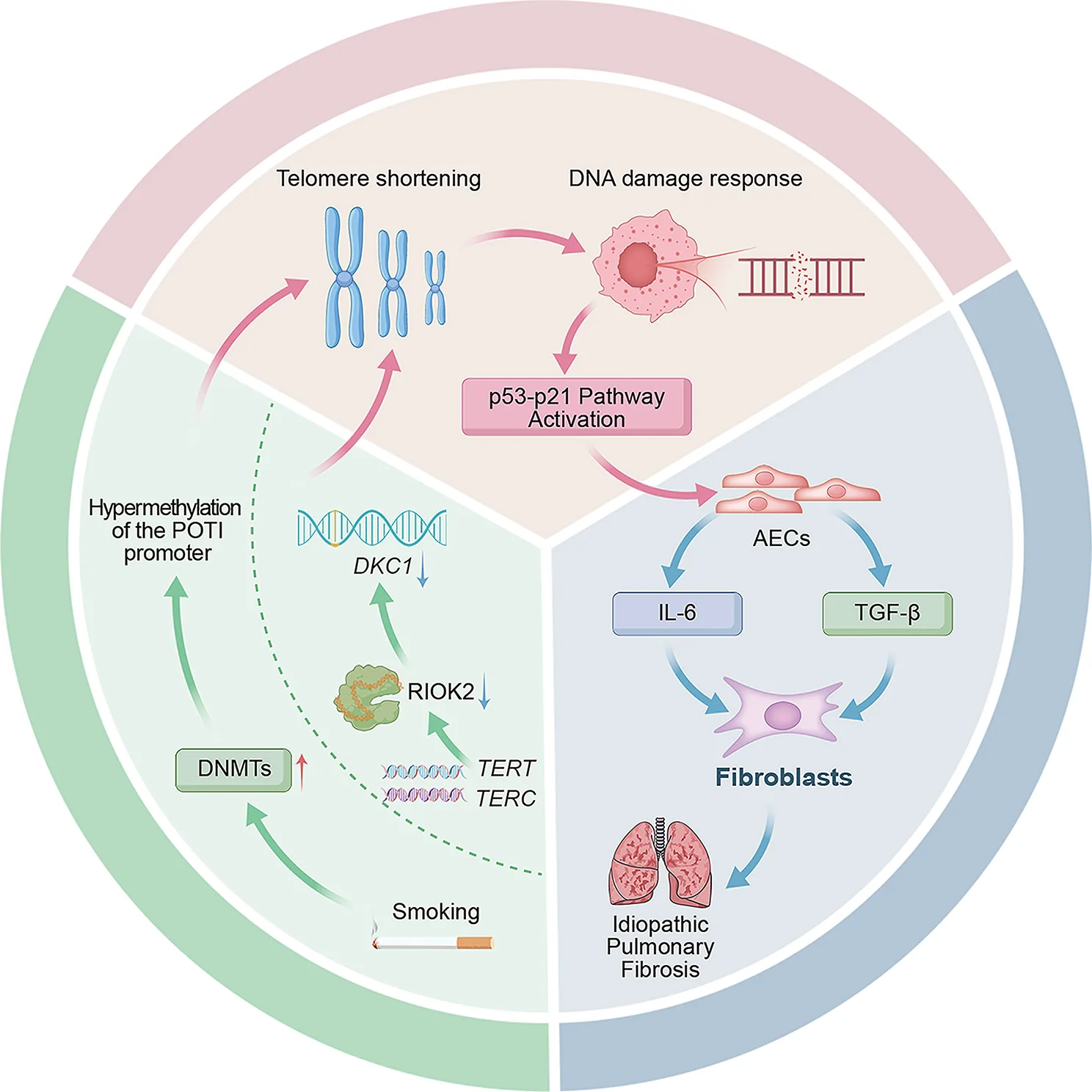
Interplay of telomere dysfunction and cellular senescence in IPF

Cellular senescence is an important mechanism by which the body protects itself against many pressures. It is characterized by permanent cell-cycle inhibition and changes in metabolic and secretory functions. Telomeres are repetitive DNA sequences found at the ends of chromosomes. They gradually shorten with each cell division and act as a key biomarker of cellular aging [26]. Maintaining a balance in telomere length is crucial for the aging process. When telomeres reach a critical short length threshold, the DNA damage response (DDR) triggers, pushing cells into an irreversible aging state. Telomere shortening is confirmed as the core pathological basis for the development of IPF [27]. As illustrated in Fig. 2, which summarizes the molecular pathways linking telomere dysfunction, cellular senescence, and fibrotic progression. Genetically, defects in telomere maintenance genes are early pathogenic factors in the development of IPF. Mutations—whether germline or somatic—in *TERT*, *TERC*, or dyskeratosis congenita 1 (*DKC1*) can directly weaken telomerase complex function. This accelerates telomere shortening and increases the risk of IPF [28]. Recent studies show that when telomere POT1 is downregulated, telomere dysfunction and aging of AECs are promoted [29]. Senescent AECs secrete a senescence-associated secretory phenotype (SASP). SASP includes interleukin-6 (IL-6) and transforming growth factor-beta (TGF-β). These factors activate fibroblasts through paracrine signaling and increase the transcription of extracellular matrix ECM genes, such as collagen [30, 31]. The transcription factor RIOK2 binds the promoters of *DKC1* and other telomerase-related genes. When RIO kinase 2 (RIOK2) is absent, telomerase components like *DKC1* are expressed less, worsening telomere shortening in lung fibroblasts [32]. These genetic defects disrupt the dynamic balance of telomere length, further activating DDR. This drives cell-cycle arrest via the p53-p21 pathway and maintains the senescent state of AECs. Multiple regulatory mechanisms interact in this process. Environmental factors, such as smoking, can worsen telomere dysfunction through epigenetic mechanisms. For example, upregulation of DNA methyltransferases (DNMTs) blocks transcription by causing hypermethylation of the POT1 promoter. An imbalance in histone modifications can also regulate profibrotic gene expression [29]. Telomere mutations determine innate susceptibility to IPF. Recent GWAS finds PCSK6 variants do not raise disease risk but worsen epithelial senescence and fibrosis. In vitro experiments reveal dual functions of PCSK6. Its protease activity induces epithelial proliferation independent of PAR-1 to trigger fibrosis. Secreted PCSK6 activates MMP/MMP14 via paracrine signaling to degrade collagen. Yet its profibrotic epithelial injury dominates and offsets collagen-protective effects. This mechanism explains why high PCSK6 correlates with poor IPF prognosis [33, 34].

### Immune microenvironment dysregulation and mucosal defense defects

**Fig. 3:**
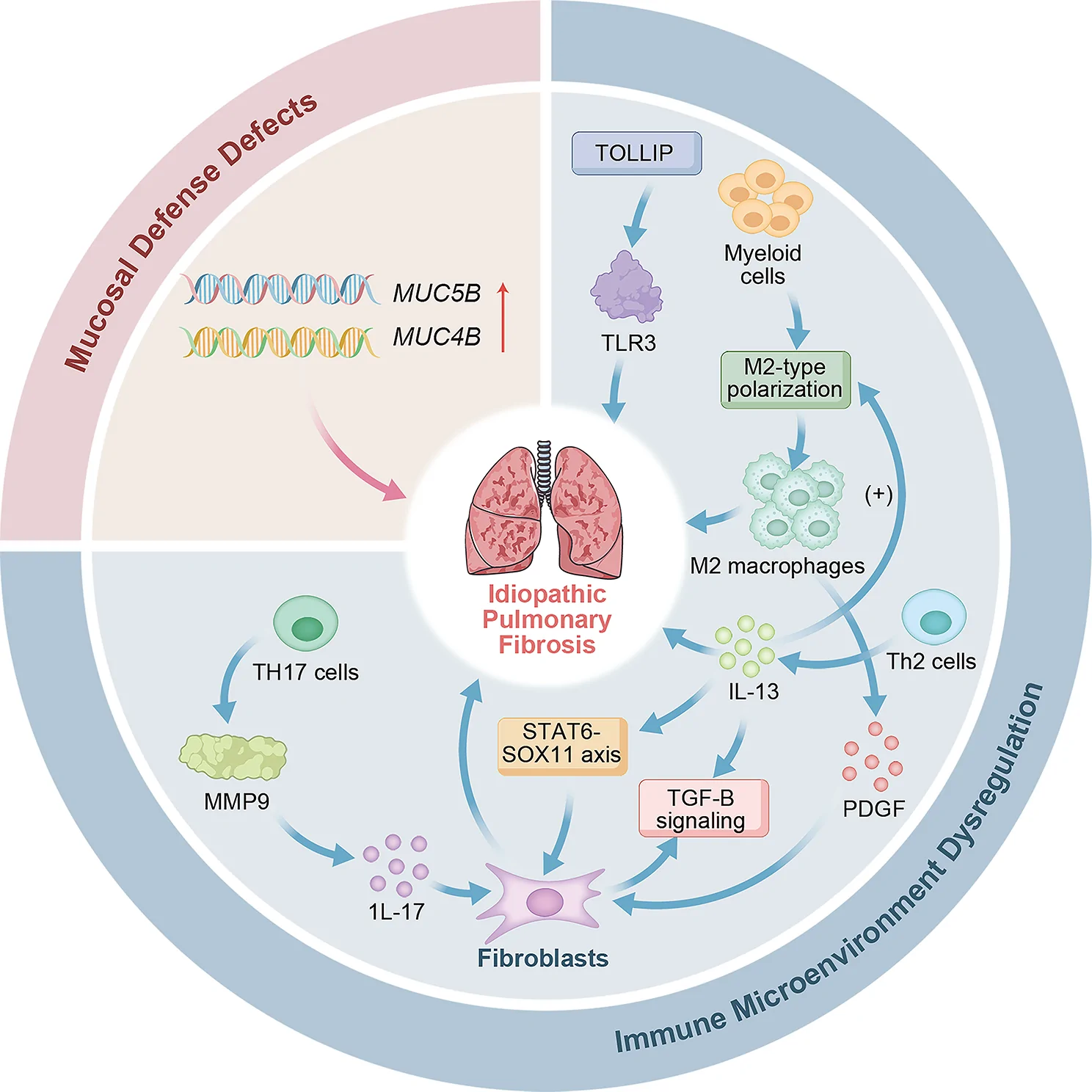
Mechanistic overview of mucosal and immune dysregulation in IPF

In the pathogenesis of IPF, genes related to mucosal defense play a crucial role. Studies have shown that genes such as *MUC5B* and Mucin 4B (*MUC4B*) are highly expressed in lung tissue from IPF patients. This abnormal expression has important pathological significance in the disease’s development [35]. Figure 3 illustrates that *MUC5B* is especially expressed in the distal airways and honeycomb cyst regions of IPF patients’ lungs. High *MUC5B* expression is associated with dysfunction of mucus-ciliary clearance [36]. Normally, the mucociliary system keeps the airways stable by clearing bacteria and other foreign particles. Excessive *MUC5B* alters the properties of mucus, making it thicker and slower to move. This change hinders the body’s ability to clear mucus and harmful substances. The Toll Interacting Protein (*TOLLIP*) gene also plays a central role in IPF. TOLLIP acts mainly through the Toll-like receptor (TLR) and TGF-β signaling pathways. Changes in the *TOLLIP* gene make Toll-like receptor 3 (TLR3) overactive and disrupt immune system balance. This leads to persistent inflammation after lung tissue loss and fosters fibrosis. Gene variation also reduces TOLLIP’s usual inhibition of the profibrotic TGF-β signal, increasing the risk of fibrosis. Both mechanisms contribute to the onset of IPF and to its progression [12, 36]. Disruption of the pulmonary immune microenvironment is another key mechanism. Polarization of macrophages and imbalance of T cell subsets are important factors. Myeloid cells, under signals from the lung microenvironment, undergo significant M2-type polarization. This is evidenced by the enrichment of CD163 + macrophages [37].

Resident alveolar macrophages exhibit microenvironment-dependent M1/M2 phenotypic plasticity. Imbalanced macrophage polarization maintains the secretion of profibrotic TGF-β and PDGF, impairs ECM remodeling and lung structure, and promotes IPF progression [38]. Fibrotic lungs present drastic macrophage phenotypic reprogramming, with reduced alveolar FABP4 macrophages and accumulated interstitial SPP1 macrophages that correlate with poor patient prognosis. Elevated SPP1 activates JAK2/STAT3 signaling to induce M2 macrophage polarization, which upregulates CD206 and secretes TGF-β and IL-10. Focal SPP1 M2 macrophages drive pulmonary fibrosis via paracrine TGF-β. They trigger epithelial-mesenchymal transition (EMT) in alveolar epithelial cells and fibroblast-myofibroblast transformation, thereby increasing COL1A1-mediated collagen deposition and causing irreversible lung structural damage [39, 40]. Disruption of T cell subset balance also occurs as a Th17/Th2 imbalance. Effector factors secreted by Th17 cells, such as IL-17, play dual roles in IPF. This inflammatory loop further accelerates IPF, especially in acute exacerbation [41–43]. Interleukin-13 (IL-13) from Th2 cells enhances IL-13 signaling sensitivity. It does this by downregulating IL13RA2 expression in fibroblasts. The STAT6-SOX11 axis guides this process. It changes fibroblasts into other cell types and activates fibrotic genes such as Periostin. This axis also promotes the M2-type macrophage switch and activates dormant TGF-β1 via matrix metalloproteinase-9 (MMP-9). These pathways accelerate IPF by directly and indirectly activating TGF-β1 [44]. In brief, Fig. 3 illustrates the synergistic network between mucosal defense defects and immune dysregulation in IPF. This integrated mechanism—driven by genetic variants, impaired clearance, and cell polarization—elucidates disease progression and highlights potential targets for precision therapy.

### Remodeling and aberrant mechanosensing

**Fig. 4:**
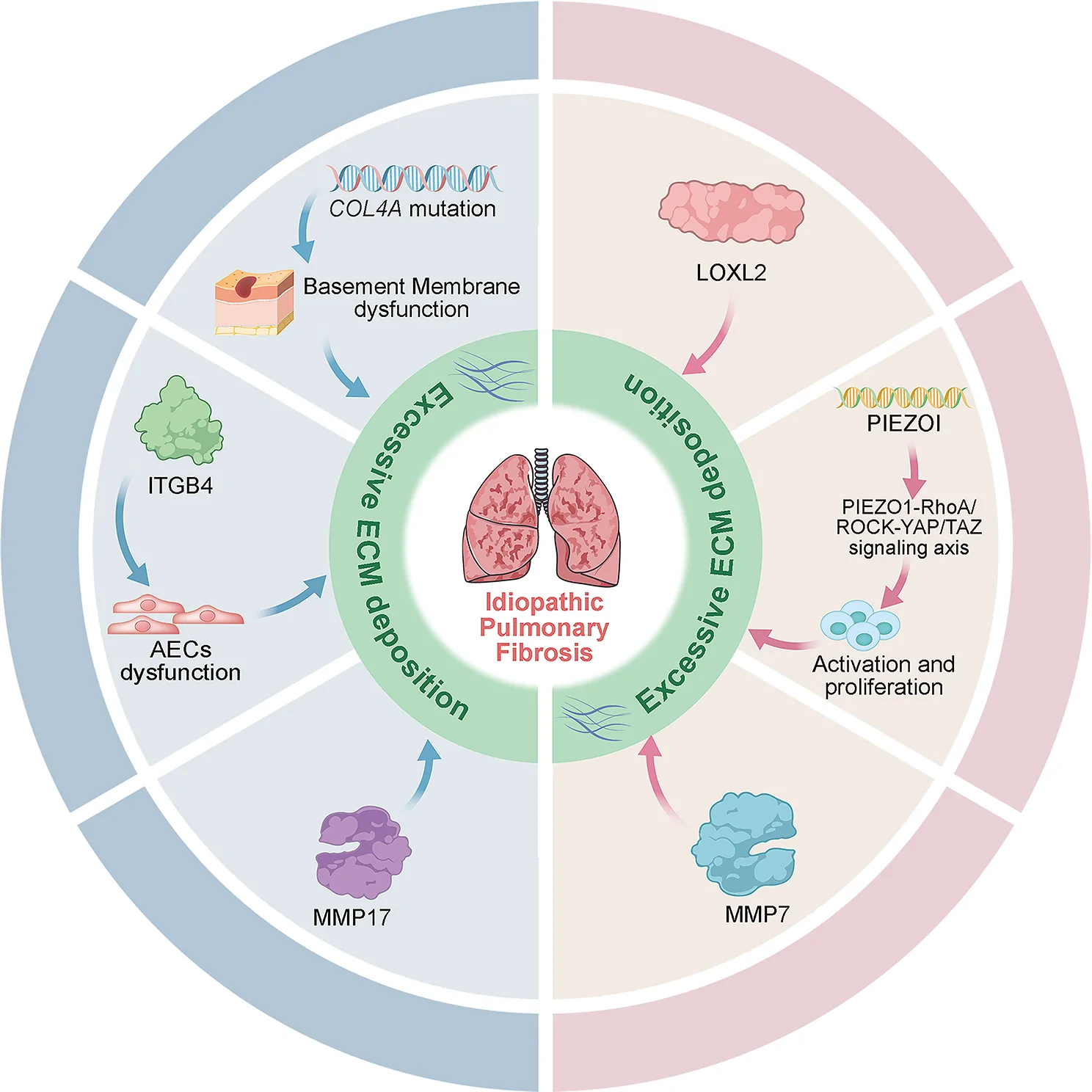
Matrix remodeling and mechanotransduction in IPF

In the pathological evolution of IPF, alterations in mechanical stress induced by matrix remodeling are highly associated with metabolic stress pathways. These two processes synergistically regulate fibroblast activation and proliferation, collectively accelerating the progression of the disease **(**Fig. 4).Mechanical stress changes induced by matrix remodeling are closely associated with the metabolic stress pathway. Both work together to accelerate the development of IPF by regulating fibroblast activation and proliferation. It is known that people with IPF have too much ECM in their lungs, which destroys the structure of lung tissue and changes its mechanical qualities. This process occurs when many cellular and molecular pathways interact in complex ways. This causes an imbalance in matrix remodeling and an abnormal perception of mechanical stress [36, 45]. As shown in Fig. 4, these changes destabilize the triple-helical structure of type IV collagen, leading to the breakdown of the Basement Membrane (BM). This collapse increases the BM’s susceptibility to proteolytic degradation and compromises its essential function as a scaffold for cellular adhesion. As a result, the integrity of the alveolar-capillary interface is undermined [46]. Alongside this structural decay, Piezo-type mechanosensitive ion channel component 1 (PIEZO1) becomes highly expressed in Postn⁺ myofibroblasts. When PIEZO1 senses membrane stress, it facilitates calcium entry into the cell, which in turn activates fibroblasts, driving their proliferation and ECM production through the PIEZO1-RhoA/ROCK-YAP/TAZ signaling axis. Importantly, both genetic deletion of PIEZO1 and pharmacological inhibition with GsMTx4 have been shown to reduce fibrogenesis in murine models [45]. Additionally, mutations in the integrin ITGB4 impair cell-matrix adhesion, diminishing AECs’ capacity to sense physiological mechanical cues and triggering maladaptive repair responses [47, 48]. Further supporting the role of matrix remodeling, gene expression profiling from 2002 revealed that matrix metalloproteinases (MMPs) are crucial in IPF at the proteomics level. In particular, matrix metalloproteinase-7 (MMP-7) is highly expressed in lung cells from people with IPF, and its deficiency in mice alleviates cirrhosis. MMP7 is involved in both ECM degradation and reconstruction, contributing to lung structural damage and fibrosis. These findings form a strong basis for further exploration of MMP7 as a potential disease biomarker and therapeutic target [49]. Furthermore, rare variations in matrix metalloproteinase-19 (MMP-19) have been identified as a genetic risk factor for IPF. The pathogenic mechanism of MMP-19 is not merely increased expression; rather, it is more often caused by pathogenic mutations that lead to loss or dysfunction of MMP-19 protein function, thereby preventing it from exerting its role in inhibiting fibrosis. This ultimately disrupts the balance of lung tissue repair and exacerbates fibrosis progression [50]. In patients with IPF, the abnormally high expression of lysyl oxidase-like protein 2 (LOXL2) promotes the cross-linking of collagen and matrix hardening, and activates TGF-β, forming a self-perpetuating malignant cycle from matrix remodeling to continuous fibrosis [51].

### Metabolic stress and synergistic signaling

**Fig. 5:**
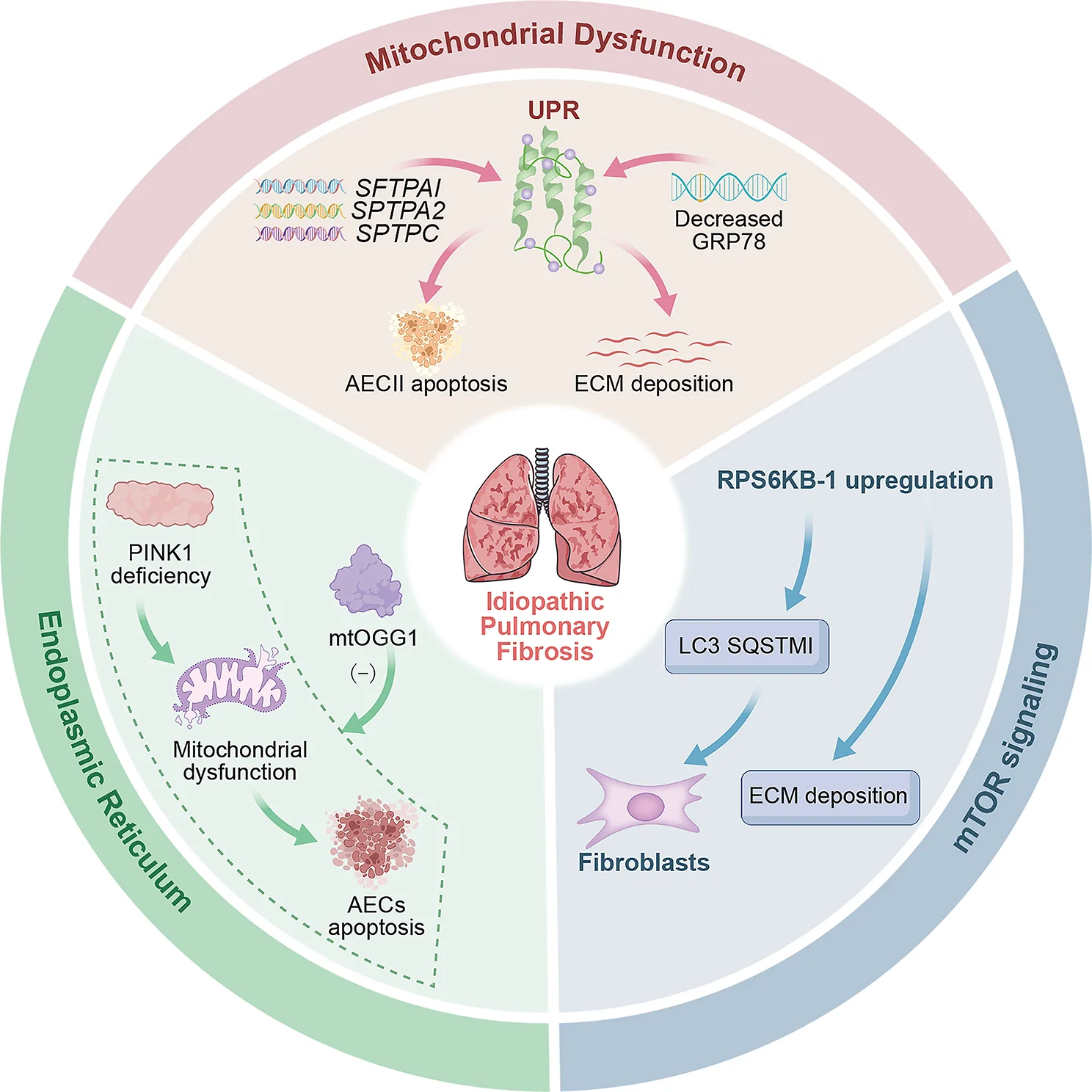
Synergistic metabolic stress pathways in the pathogenesis of IPF

IPF results from multiple pathogenic processes. Building on Section “Remodeling and aberrant mechanosensing”, we focus on intracellular metabolic stress pathways, which synergistically drive fibrosis. As shown in Fig. 5, key factors include mitochondrial dysfunction, endoplasmic reticulum stress (ERS) due to protein misfolding, and overactive mTOR signaling.Mitochondrial dysfunction, a major contributor, arises from PTEN-induced kinase 1 (PINK1) deficiency, which impairs mitophagy and leads to AECs apoptosis [52]. Oxidative stress further exacerbates mitochondrial damage, while mitochondrial 8-oxoguanine DNA glycosylase 1 (mtOGG1) preserves mtDNA integrity and mitigates AEC injury. It repairs AEC damage caused by PINK1 deficiency and oxidative stress. This prevents the progression of IPF [53].Besides mitochondrial dysfunction, ER stress and impaired surfactant protein metabolism also drive alveolar epithelial injury and fibrosis, illustrating the synergy of cellular stress pathways in IPF.

Building on the discussion of cellular stress, ERS, and surfactant metabolism genes, these factors also play a key role in IPF. Studies show that mutations in the *SFTPC* gene, which encodes lung surfactant protein C (SP-C), and the *SFTPA2* gene, which encodes surfactant protein A (SP-A), are closely linked to familial pulmonary fibrosis (FPF) and IPF [54]. Thomas et al. [55] first established that heterozygous (involving only one copy of a gene) mutations in *SFTPC* are associated with adult usual interstitial pneumonia (UIP) and pediatric cellular non-specific interstitial pneumonia (NSIP). These mutations cause pro-protein misfolding and lead to toxicity in type II alveolar epithelial cells (AECII). This establishes a direct connection between genetic defects and epithelial injury. Similarly, mutations in *SFTPA2* result in endoplasmic reticulum (ER) retention of misfolded proteins. This underscores the critical role of the unfolded protein response (UPR)—a cellular stress response related to the ER—in mediating cellular damage [56]. This profibrotic cascade is initiated by SFTPA1 mutations. Misfolded mutant SFTPA1 is retained in the ER to trigger ERS and UPR, selectively activating the IRE1α-JNK axis. Persistent JNK phosphorylation upregulates necroptosis effector RIPK3, which drives inflammatory AECII death via RIPK3-MLKL, while apoptotic caspase-3 remains unchanged. In Sftpa1-KI mice, JNK inhibition reduces RIPK3 and fibrosis, yet RIPK3 overexpression reverses this protection; Ripk3/Mlkl knockout alleviates epithelial necroptosis and lung fibrotic lesions. Together, these data define the full pathway: mutant SFTPA1 → ER stress → IRE1α-JNK → RIPK3-MLKL-mediated AECII necroptosis [57]. GRP78 is an ER chaperone protein. It is also known as 78-kDa glucose-regulated protein. GRP78 helps maintain protein balance in the ER. If GRP78 is reduced, misfolded proteins accumulate, worsening ER stress. This activates TGF-β (transforming growth factor beta) signaling and speeds ECM production and lung remodeling [58]. These examples show the effects of disrupted proteostasis. Biallelic *ABCA3* mutations directly impair lipid transport and induce early-onset severe interstitial lung disease, representing well-established genetic drivers of IPF. Cohort studies in Chinese populations have validated the heterozygous missense variant p.G1205R as a susceptibility marker for sporadic interstitial lung disease in adults. This variant disrupts exonic splicing enhancer motifs and reduces the production of functional ABCA3 protein, leading to impaired lipid transport and toxic lipid accumulation in type II alveolar epithelial cells, which subsequently trigger endoplasmic reticulum stress and epithelial apoptosis. Damaged epithelial cells secrete abundant profibrotic signals to activate the TGF-β cascade and initiate fibrogenesis. Furthermore, such genetic metabolic defects can interact with risk factors including aging and autoimmune disorders to accelerate the progression of pulmonary fibrosis [59, 60].

There is a mechanism called mTOR that controls cell growth and metabolism. At different stages of IPF, it works in an unusual way [19]. This includes mTOR and RPS6Kβ-1, a ribosomal protein S6 kinase. Their increase promotes fibroblast growth and excess ECM, which drives pulmonary tissue fibrosis [61]. In mouse IPF models treated with bleomycin and in human fibroblasts treated with TGF-β1, the mTOR pathway is activated. Increased mTOR phosphorylation blocks fibroblast autophagy. It also boosts fibroblast activation, proliferation, migration, and excess ECM protein. These changes speed up IPF [62]. More research shows that the mTOR pathway controls genes involved in autophagy. Autophagy is important for maintaining cellular balance and may help prevent fibrosis. During IPF, excessive mTOR activity suppresses autophagy genes, such as microtubule-associated protein 1 light chain 3 and sequestosome 1. This disrupts autophagosome formation and breakdown. As a result, inflammation is triggered, and fibrosis is promoted. Weak autophagy increases lung tissue damage and boosts fibroblast activation and collagen production [36]. A GWAS study shows that mTOR plays a role in IPF. A GWAS study of five groups identified several gene loci associated with increased risk of IPF. Some genes at these sites are in the mTOR pathway. Through mTORC1 signals, these genes control cell growth rates. These results support mTOR’s important genetic role in IPF [19].

## Precision therapeutic strategies: clinical applications based on genetic stratification

The multifaceted pathogenesis of IPF—encompassing senescence, telomere attrition, immune dysregulation, and matrix-metabolic stress—underscores the necessity for targeted interventions. Current clinical efforts are advancing beyond traditional drugs toward gene editing and cell therapies. This section evaluates the clinical applications, outcomes, and challenges of these evolving therapeutic strategies.

### Clinical efficacy and limitations of approved pharmacological therapies

Nintedanib and pirfenidone are the two main drugs approved for the treatment of IPF [9, 63]. They delay disease progression through different mechanisms. Nintedanib exerts anti-fibrotic effects by inhibiting tyrosine kinase receptors, such as VEGFR, FGFR, and PDGFR. It interferes mainly with the FAK/ERK/S100A4 pathway. Nintedanib inhibits migration and proliferation of pulmonary fibroblasts and reduces production and deposition of collagen I, collagen III, and α-SMA. In animal and cell experiments, nintedanib significantly slows lung function decline. This is verified by its inhibition of TGF-β1-induced proliferation and migration of HFL-1 cells. Overexpression of FAK partially reverses nintedanib’s effect and slows the fibrotic process [64, 65]. Pirfenidone’s anti-fibrotic action primarily results from inhibition of the TGF-β pathway. This reduces the conversion of fibroblasts into myofibroblasts and suppresses collagen and ECM accumulation. Pirfenidone also has anti-inflammatory effects. It reduces the expression of tumor necrosis factor-alpha (TNF-α), interleukin-4 (IL-4), and IL-13. Regulating the immune response, it inhibits proliferation of dendritic cells and T cells. It also inhibits B-cell-induced activation of fibroblast-like cells [66]. The clinical effects of nintedanib and pirfenidone are good, but their applications, efficacy, and side effects vary among individuals. Skin problems and stomach pain are common side effects of nintedanib. Pirfenidone, in the same way, can cause skin and stomach effects, especially at higher doses. Though these may affect patient compliance, side effects are mostly mild to moderate and manageable with dose adjustment, temporary discontinuation, or symptomatic treatment [67, 68]. Overall, nintedanib and pirfenidone mark significant progress in anti-fibrotic treatment. They allow IPF patients to delay functional decline and improve prognosis. Although not a cure for IPF, they slow disease progression [63]. The full follow-up analysis of phase III FIBRONEER-IPF trial confirms that nerandomilast, a selective PDE4B inhibitor, significantly slows FVC decline in IPF patients [69]. In vitro studies verify that treprostinil exerts unique antifibrotic effects via dual pathways mediated by the IP receptor. The phase 3 TETON trials are investigating the long-term lung-protective efficacy of inhaled treprostinil in IPF [70, 71].

### Emerging targeted therapies in phase II/III clinical trials

In recent years, new targeted therapies for IPF have advanced to phase II/III clinical trials (Table 1). Telomere-targeted therapy is a focus. Danazol has shown positive results in clinical studies for IPF. Its main mechanism is delaying telomere shortening, thereby protecting chromosomal stability and slowing IPF progression [72]. Clinical trials show that danazol modulates telomerase activity by regulating sex hormones. This improves the IPF linked to telomere maintenance gene mutations. Danazol may slow lung function decline in early stages but is less effective in advanced IPF [73, 74]. In the III-stage STARSCAPE study, zincpentraxin alfa, another targeted drug, did not work significantly better than a placebo. Even though past studies looked good, this happened. Zinpentraxin alfa is a pentraxin-2 variant. In preclinical tests, it was shown to reduce fibrosis. This is likely because it alters the function of macrophages and fibroblasts. However, in the III-stage trial, zinpentraxin alfa did not outperform placebo on key endpoints, such as FVC and 6MWD [75]. The trial was prematurely terminated per prespecified interim futility analysis, and the promising efficacy signal observed in its phase II trial was actually driven by outliers with extreme FVC decline in the placebo arm, which reveals the methodological flaw that small-sample phase II trials relying merely on mean values for efficacy assessment tend to generate false-positive results. Collectively, these findings confirm that single-target intravenous protein agents solely modulating the monocyte-macrophage pathway fail to improve pulmonary function in IPF patients; besides, multiple trial design factors including high intra-patient variability of 6MWD, heterogeneous baseline characteristics of participants, and the imputation strategy for missing FVC data of deceased patients can substantially interfere with efficacy interpretation in IPF clinical trials. Targeting interleukin-11 (IL-11) is a new approach. Therapies against IL-11 target the IL-11 receptor α subunit/glycoprotein 130 complex. They specifically inhibit the JAK2/STAT3 and ERK pathways. This stops fibroblast stimulation, as shown by ACTA2 expression, and the SASP caused by p16 and p21. By targeting these processes, we hope to reduce remodeling of lung tissue and the buildup of ECM involving COL1A1 and FN1. Preclinical models show that fibrosis can be reversed. From lab tests to early trials on people, clinical development is moving forward. The full efficacy and safety profile still needs more evidence [76–78]. The JAK/STAT pathway is controlled by cytokines such as IL-4, IL-6, IL-13, and TGF-β. JAK inhibitors bind to ATP-binding sites and stop the process. This blocks the inflammatory microenvironment triggered by M1 macrophages. It halts the fibrotic process driven by M2 macrophages and fibroblasts. Studies show that tofacitinib improves 6-month survival in early anti-MDA5-positive amyotrophic lateral sclerosis patients, increasing it from 78% to 100%. In studies of systemic sclerosis-related ILD, 93.5% of patients showed no progression after JAK inhibitor therapy. This supports the promise of JAK inhibitors as dual anti-inflammatory and anti-fibrotic treatments. Although promising, large randomized controlled trials are still needed to confirm their effectiveness and define their clinical role [79, 80]. The LPA1 receptor antagonist blocks LPA from binding its receptor. It inhibits fibroblast recruitment, activation, proliferation, and survival. It also reduces AEC apoptosis and vascular leakage, interfering with IPF pathology at many levels [81]. First-generation LPA1 antagonists caused liver and gallbladder toxicity due to off-target effects. However, the second-generation antagonist admilparant slowed the decline in lung function in IPF and progressive pulmonary fibrosis (PPF) patients during Phase II trials. The decline rate in PPF patients dropped by 54% and 74%. Admilparant also showed good safety and tolerability. It is now in global Phase III development [82, 83]. The PDE4B inhibitor Nerandomilast attenuated FVC decline in a phase III trial for IPF, mechanistically via inhibition of the NLRP3 inflammasome and TGF-β/Smad signaling, conferring both anti-inflammatory and anti-fibrotic effects [69]. In the phase II trial (PRAISE), pamrevlumab significantly slowed the decline in FVC in patients with IPF and improved quantitative HRCT fibrosis scores, demonstrating promising antifibrotic potential [84, 85]. However, in the phase III ZEPHYRUS-1 trial, the primary endpoint of change in FVC was not significantly different from placebo (between‑group difference 70 mL, *P* = 0.29), and no benefits were observed in secondary endpoints or patient‑reported outcomes [86]. The drug thus faced a development setback due to failure to replicate the positive phase II results in a confirmatory trial, which may be related to differences in baseline patient characteristics and the allowance of concomitant use of approved antifibrotic agents.

**Table 1:** Emerging targeted therapies for IPF categorized by mechanistic clusters

Mechanism Target	Pharmacological Agent	Company	Clinical Trial Identifier	Clinical Phase	Key Clinical Outcomes
Telomere targeting	Danazol [72–74]	Sterling-Winthrop	NCT01441037 NCT03312400 NCT05012111	Phase II	Efficacy in attenuating IPF progression remains inconclusive; therapeutic adherence is limited by adverse event profiles.
Antifibrotic	Zinpentraxin alfa [75]	F. Hoffmann-La Roche, Ltd.	NCT04552899	Phase III	Trial terminated prematurely following interim futility analysis.
Anti-IL-11	9MW3811 [78] BI 765,423 [78] LASN01 [78]	Mabwell Boehringer Ingelheim Lassen Therapeutics	NCT05801367 NCT05432596 NCT05331300	Phase I/II	Favorable safety profile with no serious adverse events reported; FVC evaluation is currently ongoing.
JAK inhibition	Tofacitini [79, 80] Baricitinib [80] Ruxolitinib [80]	Pfizer Eli Lilly & Incyte Incyte & Novartis	NCT03274076 NCT04035668 NCT03685513	Phase II	Demonstrates significant potential as a dual-action “anti-inflammatory and antifibrotic” therapy.
LPA1 antagonism	BMS986020 [81] Admilparant [82, 83]	Bristol-Myers Squibb Bristol-Myers Squibb	NCT01766817 NCT04308681	Phase II/III	Second-generation antagonists reduced the rate of ppFVC decline by 54% and 74% in II and PPF cohorts, respective.
PDE4B	Nerandomilast [69]	Boehringer Ingelheim		Phase III	The drug met its primary endpoints and demonstrated efficacy in improving lung function, as measured by (FVC).
PTGIR	Treprostinil [70, 71]	United Therapeutics	NCT04708782 NCT05255991	Phase III	16-week inhaled treprostinil significantly improved FVC by 168.5 mL (*P* = 0.011) and reduced pulmonary exacerbations (26% vs. 39%, *P* = 0.02)
CTGF/CCN2	Pamrevlumab [84–86]	FibroGen	NCT01890265 NCT01262001 NCT03955146	Phase II/III	In phase II, it significantly slowed FVC decline and improved HRCT findings; however, in phase III, the primary endpoint was not met, and the between‑group difference was not significant.

### Frontier explorations in IPF therapeutics

The pulmonary SP-C encoded by the *SFTPC* gene primarily maintains the function and stability of pulmonary alveolar surfactant. Mutations in the *SFTPC* gene can lead to dysfunction of AECs, thereby promoting the development of IPF, and play a crucial role in the pathogenesis of hereditary IPF [54]. To address this issue, the researchers employed the CRISPR-*Cas9* gene-editing technology to correct the *SFTPC*-I73T mutation in the patient-derived induced pluripotent stem cells to the wild-type (*SFTPC*-WT/WT) state, thereby restoring the normal *SFTPC* allele. This gene-editing strategy provides a new gene therapy approach for diseases caused by *SFTPC* mutations, such as familial ILD and IPF [87]. Studies in animals have shown that mesenchymal stem cells (MSCs) can successfully treat bleomycin-induced IPF, improve lung function, and modulate immune responses [88–90]. MSCs help tissues heal and slow the fibrotic process in several ways, including altering the immune system. For example, MSCs reduce inflammation by releasing cytokines like interleukin-1 receptor antagonist (IL-1RA) and interleukin-10 (IL-10). They also help epithelial cell repair and neovascularization by releasing keratinocyte growth factor (KGF) and hepatocyte growth factor (HGF). Finally, they stop collagen buildup by controlling fibroblasts and the matrix, which lowers fibrosis [88]. Adipose-derived mesenchymal stem cells (ADMSCs) have gradually become a research hotspot due to their convenient acquisition and low trauma. Studies have shown that ADMSCs inhibit the ERK signaling pathway by upregulating protein tyrosine phosphatase receptor type R (PTPRR), reduce the expression of fibrotic genes, and thus exhibit excellent anti-fibrotic effects, making them a potential alternative for the treatment of IPF [89]. However, cell therapy still faces challenges, including cell survival rates and immune rejection. To address these issues, MSC exosomes, as an innovative cell-free therapeutic strategy, inherit the repair and regulatory functions of MSCs. MSC exosomes can promote tissue repair and slow fibrosis progression through multiple mechanisms by targeting and regulating core signaling pathways, such as TGF-β and Wnt/β-catenin, in IPF. Because live cells are removed, MSC exosomes are less likely to trigger immune rejection and tumor growth, and they have significant potential for medical use [90]. In summary, research on cell therapy for IPF has progressed from validating the therapeutic potential of MSCs in animal models, to considering ADMSCs as an effective cell therapy option, and then to MSC exosomes as an emerging non-cell-based treatment strategy. These studies not only revealed the potential of different cell types for treating IPF but also provided new ideas for the future development of safe, effective, and scalable treatment strategies.

## Prognostic prediction and population risk stratification

With the continuous development of precise treatment strategies and the in-depth study of the genetic mechanisms of IPF, individualized treatment has gradually become the core of treatment strategy. The association between genotype and treatment response allows us to tailor treatment plans. However, current treatment drugs have certain limitations and cannot meet the needs of all patients. Therefore, in addition to the already marketed drugs, innovative targeted therapies and treatment methods for IPF are receiving more attention. These new therapies are based on different mechanism clusters. They want to fix the problems with current treatments and take big steps forward. At the same time, a better understanding of gene-environment interactions and genetic biomarkers is making risk stratification and prognostic prediction key in precision medicine. More accurate disease management and patient monitoring can be accomplished by applying the MGRS system. Next, we will cover the advantages and disadvantages of currently available medications. We will then discuss state-of-the-art research and the prospects of novel targeted medicines. Finally, we will emphasize the importance of gene-environment interactions in identifying those at risk for IPF.

### Clinical translation of genetic biomarkers

On the molecular level, *Tspyl2* serves as a transcriptional regulator. It suppresses the conversion of lung fibroblasts to myofibroblasts and blocks the TGF-β/Smad3 signaling pathway, exerting anti-fibrotic effects. Meanwhile, *TERT* rs2736100 (A), an IPF risk allele, shows variable protective effects against airway lesions. This is especially notable in cases involving rheumatoid arthritis (RA) [91].Several genetic markers are important for the development and progression of IPF and related ILDs because they affect immune responses, telomere stability, and cell growth. The functional variant in *TLR* rs3775291 reduces ligand binding. This leads to decreased protective IFN-γ production and weaker inhibition of fibroblast proliferation, as NF-κB and IRF3 signaling pathways are inhibited. Clinical studies show that this variation is related to accelerated decline in FVC and higher risk of death (*HR* = 4.98) [92]. The A allele of *TERT* rs2736100 is a susceptibility locus for IPF. It causes disease by reducing *TERT* expression and shortening telomere length. Patients with the CA genotype have a poorer prognosis with pirfenidone treatment. Japanese people with RA are more likely to get airway diseases if they carry the C gene (*OR* = 1.40) [93]. *ATP11A* rs1278769 (G) acts as an independent risk factor for NSIP in elderly patients with RA. It achieves this by down-regulating gene expression in the lungs (*OR* = 2.15) [94]. The *TOLLIP* rs5743890 (TT genotype) is associated with significant pathogenicity. It is linked to IPF susceptibility and high mortality rates. In drug intervention studies, the survival benefit for patients with the TT genotype showed a negative trend with longer pirfenidone treatment [93]. Finally, although *TGF-β* rs1800470 (GA genotype) promotes collagen deposition through the TGF-β/Smad pathway and worsens disease, patients with this variant and long-term pirfenidone treatment still show a significant survival association [93]. A two-stage international GWAS identified intronic variant rs35647788 of PCSK6 as a genome-wide significant prognostic locus for IPF. While this variant does not increase disease susceptibility, it confers a 4.11-fold higher hazard of death or lung transplant and shortens transplant-free survival. PCSK6 levels in lung tissue and peripheral blood can stratify patients by IPF progression risk. A registry cohort of 428 patients with fibrotic ILDs validated that this prognostic signature is exclusive to IPF. Single-cell RNA sequencing detected enriched PCSK6 expression in IPF-specific HAS1⁺ fibroblasts. Consistently, circulating PCSK6 concentrations positively correlate with quantitative CT fibrosis load, signifying progressive disease and elevated all-cause mortality [33, 95].

### Gene-environment interactions

IPF is a typical multifactorial disease. Its pathogenesis results from interactions among several factors, including genetic susceptibility, environmental or occupational exposure, lifestyle, and age [96]. Genetic studies, especially large-scale GWAS, have identified many loci associated with disease susceptibility. New research has found variants that are highly reproducible across different populations. These include *RTEL1*, which maintains telomeres; *NPRL3*, which controls epithelial homeostasis and mTORC1 signaling; and STMN3 and KNL1, which are important for cell repair and spindle assembly [19]. A lot of people think that air pollution (PM2.5, NO₂, SO₂, etc.), metal dust, wood dust, harmful particulates, and smoking are among the main factors that cause or accelerate fibrosis [97, 98]. For individuals carrying the *TERT* mutation, smoking can trigger the “accelerated aging” mechanism. The mutation reduces basic repair capacity. Smoking damages telomeres through oxidative stress and triggers the release of inflammatory genes, such as *ISG15* and *IFN-γ*, as telomeres shorten. This strong interaction between “genes and environment” can make genetic risk that would have shown up in old age appear early as severe IPF [99, 100]. Epidemiological studies have identified several “high-risk subgroups”. People aged 60 to 70 are more likely to have IPF. It doesn’t happen very often in people under 50 [101]. People who are exposed to fibrosis-causing particles, toxic dust, or dirty air for long periods are more likely to experience repeated epithelial cell damage, chronic inflammation, oxidative stress, and dysfunctional repair mechanisms [102]. Excessive mucus secretion in the distal airways from *MUC5B* promoter mutations first weakens the lungs’ ability to remove harmful substances, such as smoke and particulates. This allows toxic factors to remain in the body and continually damage the AECs [103, 104]. For people who also have problems with their telomeres (like *TERT* or *TERC*), this ongoing damage speeds up the aging of lung type II cells and their ability to repair themselves. Stress in the environment can also make protein misfolding caused by gene variants, such as *SFTPC*, worse. This triggers stress circuits in the ER and promotes EMT. These interconnected pathways shift the normal wound-healing process towards a progressive fibrotic pathway [103]. A complex gene-environment (G×E) interaction describes how genes and environmental factors interact. Genetic variations can make a person more or less vulnerable to environmental damage. Environmental stressors can hinder tissue healing and change tissue structure through oxidative stress, epithelial damage, chronic micro-inflammatory responses, or epigenetic changes [105, 106].

### MGRs systems for clinical risk stratification

Genetic factors have long been recognized as a key component of IPF susceptibility. The classic high-penetrance single-gene variants in FPF, such as telomerase/telomere maintenance-related genes, including *TERT*, *TERC*, poly(A)-specific ribonuclease (*PARN*), and *RTEL1*, have been repeatedly confirmed to be closely associated with disease. These variations have also been detected in some sporadic IPF cases [23]. Furthermore, many studies have constructed prognostic models for IPF based on gene expression data using statistical methods. First, binary Cox regression and the Least Absolute Shrinkage and Selection Operator (LASSO) method were used to choose the core genes. After that, statistical methods such as weighted gene co-expression network analysis (WGCNA) were added to the predictive model to further improve it. This model verified its predictive ability by comparing survival differences between high- and low-risk groups, and it was found that the survival prognosis of patients in the high-risk group was poorer [107, 108]. Most people with sporadic IPF do not have a hereditary predisposition to the disease, explained by a single high-risk variation. Such a complicated genetic background may result from multiple common low-effect variants that synergistically increase genetic vulnerability. In recent years, researchers have developed the MGRS model to assess the cumulative effect of common genetic variations with small effects. The MGRS model significantly improves the risk prediction accuracy for IPF, interstitial lung abnormalities (ILA), and other diseases by integrating the effects of multiple genetic variants. In particular, recent studies have further combined MGRS with the analysis of rare variations, revealing that the “common variation - rare variation” two genetic mechanisms may correspond to different clinical subtypes: for some patients, the pathological process is mainly driven by a few highly penetrant variations, while for another group of patients, it may be caused by the cumulative effect of multiple common low-effect variations [23].

## Discussion and future perspectives

### Alignment analysis between mechanistic clusters and therapeutic strategies

**Fig. 6:**
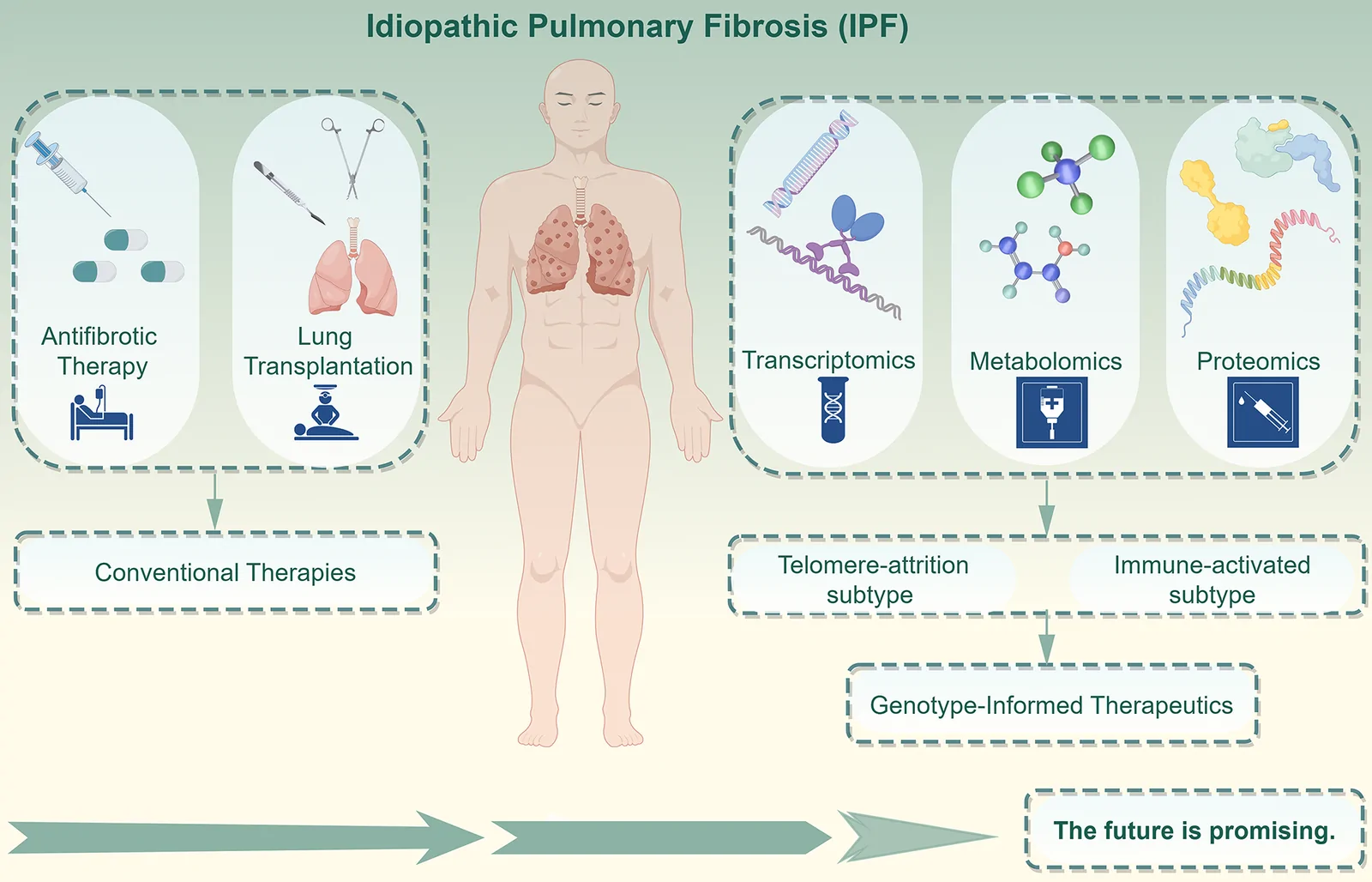
The paradigm shift in research frameworks towards a precision medicine future for IPF

Current anti-fibrotic treatments, whether approved or in research, do not fully align with the multi-mechanism nature of IPF. IPF progression is not driven by a single pathway but by coordinated signaling networks. The TGF-β pathway is central to fibrosis, promoting AECs apoptosis and EMT, encouraging fibroblast-to-myofibroblast differentiation, and excessive ECM deposition that stiffens lung tissue [109]. In addition, studies show that the Wnt/β-catenin pathway is involved in IPF, possibly working in concert with TGF-β/Smad to worsen fibrosis [110]. Various cell types—epithelial, fibroblast, myofibroblast, and immune—influence the fibrotic microenvironment via signaling pathways such as mTOR, ROS, JAK/STAT, PI3K/Akt, MAPK, and NF-κB. NcRNAs may be new regulatory points or biomarkers, attracting attention [111]. Targeted medication development increasingly targets core pathways. Reversing cell aging or TGF-β pathways can stop the cycle of “fibrosis, aging, and matrix remodeling”, slowing or reversing IPF progression [112]. TGF-β suppression reduces fibroblasts’ profibrotic signals and proinflammatory activity, as shown by single-cell RNA sequencing (scRNA-seq). Targeting both TGF-β and Wnt yields promising results. Complex system interconnections and medication side effects limit the therapeutic utility of this knowledge [111, 113, 114]. The “mismatch” between illness mechanisms and medications exists. Nintedanib and Pirfenidone delay fibrosis but do not improve some patient subgroups by inhibiting PDGFR and FGFR. The new telomere-targeting drugs aren’t very effective because they only target gene mutations and don’t address how the immune system and matrix repair work together. Treatment is not naturally tailored to each patient, so results are not always the same. Putting together microenvironmental, genetic, epigenetic, and molecular data makes it easier to predict and treat diseases. The prognosis depends on lung function, imaging, and symptoms like coughing or difficulty breathing [115, 116]. The BRP model predicts transplantation survival better than the GAP model by incorporating imaging, blood, and lung function data [115]. Finally, much has been done in IPF genetics, mechanisms, treatment development, and prognosis. Still, many challenges remain. Future research on precision medicine and interdisciplinary collaboration can improve IPF treatment and care (Fig. 6).

### Current challenges

Currently, the genetic research on IPF mainly focuses on populations of European ancestry [19]. Studies that examine only one ethnicity make it difficult to generalize about susceptibility genes, allele frequencies, and impact sizes. This applies notably to Asian, African, and Latino people. Therefore, to construct a more globally applicable genetic framework, future research must include ethnic groups that have not been adequately studied [117]. The comparative study shows that one of the most representative risk variants - the SNP (rs35705950) in the promoter region of *MUC5B* - has significantly different frequencies of its minor allele among different ethnic groups. For instance, in some Asian ethnic groups and among African Americans, the frequency of this allele is significantly lower than that in European populations. This means that its predictive power for risk among non-European ethnic groups may be diminished, as its contribution from these populations may be greatly reduced [118]. Consequently, the multi-gene risk assessment tool’s global applicability is cast into doubt by its construction based on people of European heritage. This makes it possible to over- or under-estimate how genetically vulnerable different racial groups are. To avoid racial bias, researchers should consider adding more people from diverse racial and cultural backgrounds to their samples in the future. This would help make the DNA model more open to everyone. The pathogenesis of IPF is considered to be the result of a complex interaction between “genetic susceptibility and environmental triggering factors”. Disease onset and progression cannot be explained by genetic susceptibility differences alone [96, 117]. For instance, the classic *MUC5B* promoter region SNP (rs35705950) is one of the strongest common risk factors, but it only affects some patients. Additionally, environmental factors such as smoking, air pollution, occupational exposure, and chronic micro-inhalation may act as key mechanisms in triggering excessive fibrosis repair through repeated micro-damage [119]. Recent studies have underscored the significance of gene-environment and epigenetic interactions in the development of IPF. Specifically, certain genetic variations or their regulatory regions may be “activated” or have their effects amplified by epigenetic mechanisms, including DNA methylation, histone modifications, and non-coding RNA expression, in the presence of specific environmental factors (e.g., air pollution, smoking, occupational exposure), thereby promoting or accelerating fibrosis [96, 120].

To date, GWAS studies have identified 20–30 genetic loci associated with IPF susceptibility, encompassing mechanisms such as telomere maintenance, host defense, cell adhesion, signaling pathways, cell cycle, and mucin metabolism [121]. As early assessments have indicated, 90% of GWAS-identified significant loci are in non-coding areas of the genome, and their biological functions are unknown. Although some variations have undergone high-throughput functional evaluations, the connection between the sequence and its biological consequences remains weak, indicating a lack of efficient, systematic functional validation methods. This makes the rapid, large-scale verification of the biological effects of these variations still a challenge [119, 122]. In recent years, researchers have attempted to integrate GWAS, transcriptome, quantitative trait loci expression, and single-cell or spatial transcriptomic data using multi-omics methods, with the aim of prioritizing the identification of genes with causal effects. A 2025 study identified *ANO9*, *BRCA1*, and *FAM13A* as possible therapeutic targets using many methods [120].

### Future directions

Recently, a study published in the journal “*Nature Genetics*” combined spatial and single-cell transcriptomics to reveal a spatial map of fibrosis in lung tissues from IPF patients and mouse models. This study systematically depicted the spatial distribution of different cell types in the fibrotic microenvironment and the heterogeneity of signaling pathways, further integrating tissue transcriptomics and proteomics data, and analyzed the lung tissues of patients with advanced IPF at the transcriptional and translational levels, revealing the non-coding RNA regulatory network and differences in gene-protein expression, providing important basis for the identification of potential molecular markers and therapeutic targets [123]. In recent years, researchers have merged transcriptomics, proteomics, and non-coding RNAomics data to find IPF processes and molecular markers. For instance, Zheng et al. [124, 125]. analyzed transcriptional and protein expression differences in end-stage lung tissues from IPF patients and found significant differences in pathways related to immune response, cell repair, and ECM synthesis. In addition to studying the causes of illness and identifying targets, multigene research supports the development of biomarkers for early diagnosis, prognostic assessment, and tracking therapy response. Existing studies have identified differentially expressed genes (DEGs) and proteins related to IPF through multigene integration and have proposed several potential biomarkers [126]. The review article also points out that, in the future, early diagnosis of IPF should integrate serum proteins, ncRNAs, and other biomarkers and be supplemented by comprehensive analyses of imaging, pulmonary function, and clinical features. This direction will be key to early diagnosis of IPF [127]. Based on liquid biopsy technology using circulating exosomes, a new frontier for early IPF diagnosis, significant progress has been made in recent years. Using the microfluidic disc-based chip system, researchers have screened for exosomal proteins such as Krebs von den Lungen 6 (KL-6), Calpain-2 (CAPN2), and Surfactant Protein B (SP-B), and have constructed a combined diagnostic model. This model has achieved an area under the curve (AUC) of 0.936 in the diagnosis of ILD, and can effectively identify undiagnosed cases with negative serum KL-6 levels [128]. Additionally, plasma exosomal circular RNAs, such as hsa_circ_0044226, exhibited exceptionally high diagnostic efficacy (AUC of 0.991) and demonstrated significant associations with pulmonary function parameters, thereby serving as critical molecular biomarkers for predicting acute exacerbation and disease progression in IPF [129].

In the research and management of IPF, integrating dynamic monitoring techniques to assess disease progression is of utmost importance. Traditional monitoring approaches, such as pulmonary function testing and imaging, often reveal pathological changes after the disease has advanced, limiting early intervention and individualized treatment [130]. In recent years, the use of wearable devices and digital biomarkers has opened new opportunities for long-term monitoring of disease activity. Especially in the objective collection of disease activity indicators, such as cough intensity, these devices have overcome the intermittent problems of traditional methods [131]. Advanced imaging approaches, such as collagen hybridization, provide dynamic molecular monitoring of IPF [130]. Disease development can be more accurately predicted by processing data from dynamic monitoring devices using machine learning models trained on big data. For instance, analyzing the spatial features of CT scans in combination with time-series data from clinical records can provide more accurate, real-time prognostic evaluations [132].

In conclusion, The management of IPF is shifting from empirical approaches to genomics-guided precision medicine. Table 2 summarizes the key differences between conventional and genomics-based diagnostic and therapeutic models. Future research on IPF should focus on integrating and analyzing multi-omics data and deeply exploring new molecular mechanisms and potential therapeutic targets. At the same time, combined with dynamic monitoring techniques and big data analysis, it will provide new perspectives and methods for the early diagnosis, disease progression prediction, and personalized treatment of IPF. Advancements in technology, particularly in wearable devices and molecular imaging techniques, bode well for the future of IPF management and intervention. These developments will propel research and clinical practice in this area towards ever-increasing levels of precision and personalization.

**Table 2:** Comparison of Conventional and Genomics-Guided Management in IPF

Dimension	Conventional Approach	Genomics-led Precision Medicine
Diagnostic Basis	Reliance on decline in FVC and HRCT patterns (often associated with diagnostic lag).	Early molecular warning via biomarkers (e.g., circulating exosomes, non-coding RNAs).
Therapeutic Targets	Broad-spectrum anti-fibrotic agents (e.g., Nintedanib, Pirfenidone).	Targeting causal variants (e.g., MUC5B, TERT) and synergistic pathway interventions.
Prognostic Assessment	Static clinical parameters (e.g., the GAP model: Gender, Age, Physiology).	Integrated multi-omics models (e.g., the BRP model) and Polygenic Risk Scores (PRS).
Disease Monitoring	Intermittent in-hospital follow-ups (longitudinal but discrete).	Continuous dynamic monitoring (e.g., wearable devices, molecular imaging, and AI-driven analytics).
Personalization	Universal protocols with limited consideration of genetic heterogeneity.	Tailored strategies accounting for ethnic genetic diversity and gene-environment interactions.

## Data Availability

No datasets were generated or analysed during the current study.

## Abbreviations

Abbreviation: Full Term
IPF: Idiopathic Pulmonary Fibrosis
ILD: Interstitial Lung Disease
MGRS: Multi-Gene Risk Score
PF: Pulmonary Fibrosis
ECM: Extracellular Matrix
CLAD: Chronic Lung Allograft Dysfunction
AECs: Alveolar Epithelial Cells
GWAS: Genome-Wide Association Study
TERT: Telomerase Reverse Transcriptase
TERC: Telomerase RNA Component
MUC5B: Mucin 5B
SFTPA2: Surfactant Protein A2
SFTPC: Surfactant Protein C
SFTPD: Surfactant Protein D
DDR: DNA Damage Response
DKC1: Dyskeratosis Congenita 1
SASP: Senescence-Associated Secretory Phenotype
IL-6: Interleukin-6
TGF-β: Transforming Growth Factor-Beta
ECM: Cxtracellular Matrix
RIOK2: RIO kinase 2
DNMTs: DNA Methyltransferases
MUC4B: Mucin 4B
TOLLIP: Toll Interacting Protein
TLR: Toll-like Receptor
TLR3: Toll-like Receptor 3
IL-13: Interleukin-13
MMP-9: Matrix Metalloproteinase-9
BM: Basement Membrane
Piezo1: Piezo-type mechanosensitive ion channel component 1
MMPs: Matrix Metalloproteinases
MMP-7: Matrix Metalloproteinase-7
MMP-19: Matrix Metalloproteinase-19
LOXL2: Lysyl Oxidase-Like Protein 2
ERS: Endoplasmic Reticulum Stress
PINK1: PTEN-induced Kinase 1
mtOGG1: Mitochondrial 8-oxoguanine DNA Glycosylase 1
SP-C: Surfactant Protein C
SP-A: Surfactant Protein A
FPF: Familial Pulmonary Fibrosis
UIP: Usual Interstitial Pneumonia
NSIP: Non-specific Interstitial Pneumonia
GRP78: Glucose-Regulated Protein 78 kDa
AECII: Type II Alveolar Epithelial Cells
ER: Endoplasmic Reticulum
UPR: Unfolded Protein Response
TNF-α: Tumor Necrosis Factor-alpha
IL-4: Interleukin-4
IL-11: Interleukin-11
PPF: Progressive Pulmonary Fibrosis
MSCs: Mesenchymal Stem Cells
IL-1RA: Interleukin-1 receptor antagonist
IL-10: Interleukin-10
KGF: Keratinocyte Growth Factor
HGF: Hepatocyte Growth Factor
ADMSCs: Adipose-derived Mesenchymal Stem Cells
PTPRR: Protein Tyrosine Phosphatase Receptor Type R
RA: Rheumatoid Arthritis
PARN: Poly(A)-specific Ribonuclease
LASSO: Least Absolute Shrinkage and Selection Operator
WGCNA: Weighted Gene Co-expression Network Analysis
ILA: Interstitial Lung Abnormalities
scRNA-seq: Single-cell RNA Sequencing
DEGs: Differentially Expressed Genes
KL-6: Krebs von den Lungen-6
CAPN2: Calpain-2
SP-B: Surfactant Protein B
AUC: Area Under the Curve
IFN-γ: Interferon-gamma
NF-κB: Nuclear factor kappa-light-chain-enhancer of activated B cells
